# An atypical presentation of multidermatomal herpes zoster: a case report

**DOI:** 10.1186/s12245-020-00325-6

**Published:** 2020-11-30

**Authors:** Mohammed Alhayyas, Mehmood Chaudhry, Sabrina Berdouk

**Affiliations:** Sheikh Khalifah Medical City, Abu Dhabi, United Arab Emirates

**Keywords:** Herpes, Zoster, Multidermatomal, Immunocompetent

## Abstract

**Background:**

Herpes zoster (HZ) also known as shingles is a common dermatological pathology seen in the emergency department. Multidermatomal involvement is an uncommon presentation and usually is linked to immunocompromised individuals. However, it is rarely reported in the immunocompetent population.

**Case presentation:**

We report a 30-year-old Emirati male complaining of low-grade fever for 3 days, sore throat and an uncomfortable pruritic erythematous rash over his chest and back for 2 days. He was treated the day preceding his presentation in another facility for a presumed allergic reaction after taking ibuprofen. On physical examination, he was found to have exudative tonsillitis and influenza and was treated for both and discharged. He returned to the emergency department reporting increasing pain and was referred to be seen in the dermatology clinic where a biopsy was obtained, and he was discharged with a steroid topical cream. Fourteen days later, he returned to the clinic reporting crusting of the rash; the biopsy results were positive for herpes zoster. The diagnosis of multidermatomal herpes zoster was made, and he was then treated with antivirals.

**Conclusions:**

Herpes zoster can present with atypical manifestations. Multidermatomal HZ is a rare dermatological manifestation in the immunocompetent adult. It is characterised by a rash spread over two or more adjacent dermatomes. This case highlights the challenging diagnosis of this dermatological presentation.

## Background

Herpes zoster (HZ) also known as shingles is a common dermatological pathology that arises from the reactivation of dormant varicella zoster virus in the dorsal ganglia. Typically, the condition manifests as painful vesicular skin eruption preceded by 24–72 h or neuropathic pain. The vesicular rash typically is described to follow the distribution of a single sensory dermatome without crossing the midline [1]. Localised HZ—affecting a single unilateral dermatome—is the most common presentation of HZ with thoracic dermatomes (45%), cervical (23%) and trigeminal (15%) being most affected [2]. Herpes zoster affecting multiple adjacent dermatomes is known as multidermatomal herpes zoster and is a quite rare phenomenon. Furthermore, multidermatomal HZ is associated with immunosuppression and is a very rare manifestation in the immunocompetent population [3]. The purpose of our paper is to present the case of a 30-year-old immunocompetent male with a challenging presentation of multidermatomal herpes zoster with delayed vesicular eruption.

## Case presentation

A 30-year-old male presented to the emergency department with low-grade fever, sore throat and generalised malaise that started 3 days prior to presentation. He went to his primary care physician who diagnosed him as a case of a viral upper respiratory tract infection and treated him with supportive measures (ibuprofen and paracetamol). On the following day, he developed an erythematous pruritic warm raised rash on his left anterolateral chest that extended to the medial aspect of the axilla. He also noticed the same rash on his mid-back. He presented to an emergency department 12 h after that where he was evaluated and treated with the impression of an allergic reaction to ibuprofen. He was given intravenous antihistamine and hydrocortisone in the emergency department and was discharged with topical steroid cream and antihistamine tablets. The ibuprofen was also stopped on that visit. Twelve hours after that presentation, he appeared in another emergency department complaining of worsening itchiness and heat from the rash. He described the rash to be extremely itchy and uncomfortable preventing him from sleeping at night. His past medical history was negative except for gout controlled with allopurinol. He denied any previous hospitalisations, surgeries, other medications, allergies to food, substance or medications. He also denied any recent travel or positive sick contacts. He is a regular smoker with 10-year-pack history and had no alcohol or illicit drug use history.

Upon examination, he was found to be febrile (38.2 °C) and tachycardic with a heart rate of (108 bpm). He also was found to have exudate on his tonsils and bilaterally enlarged cervical lymph nodes. Otherwise, his ear, nose and throat examination was unremarkable. His respiratory and cardiovascular examinations were all normal.

**Fig. 1:**
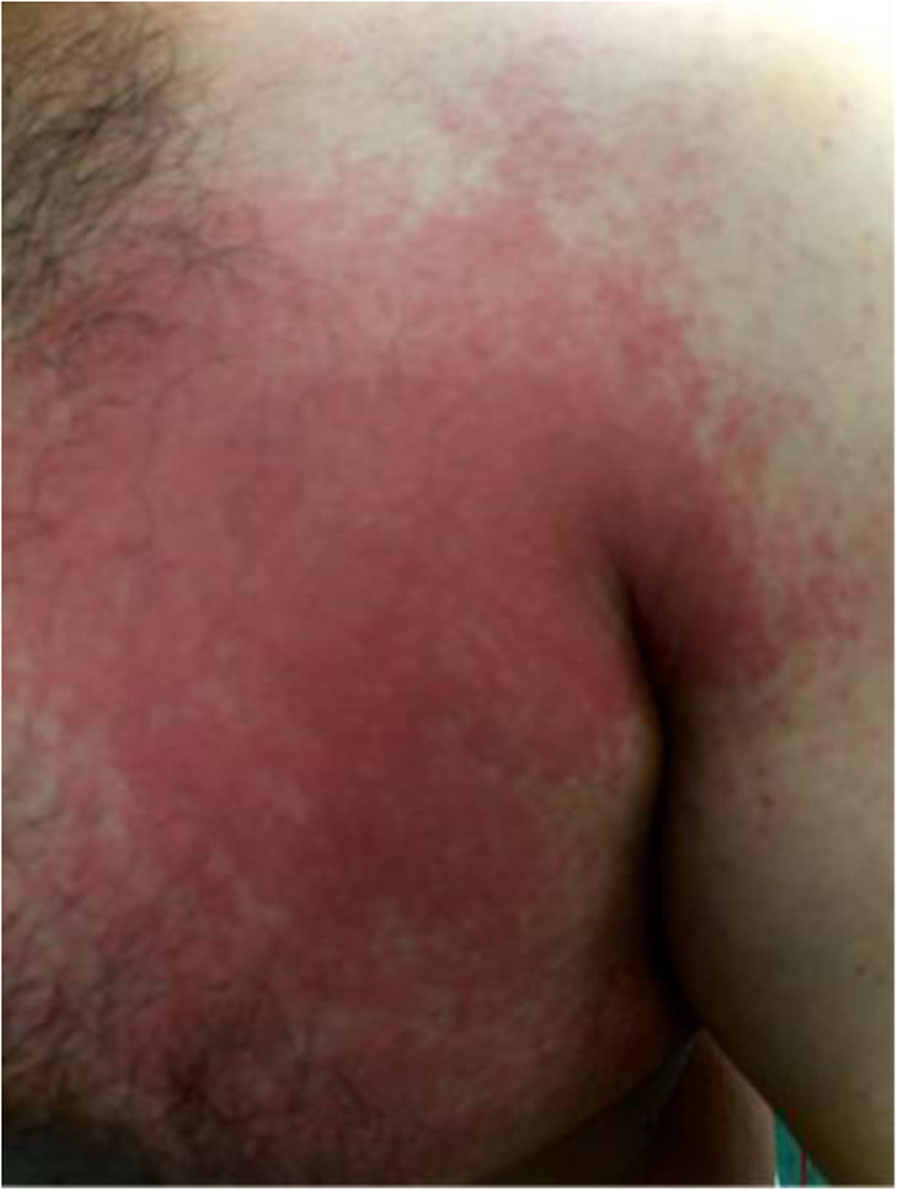
Rash on patient’s left anterior chest

**Fig. 2:**
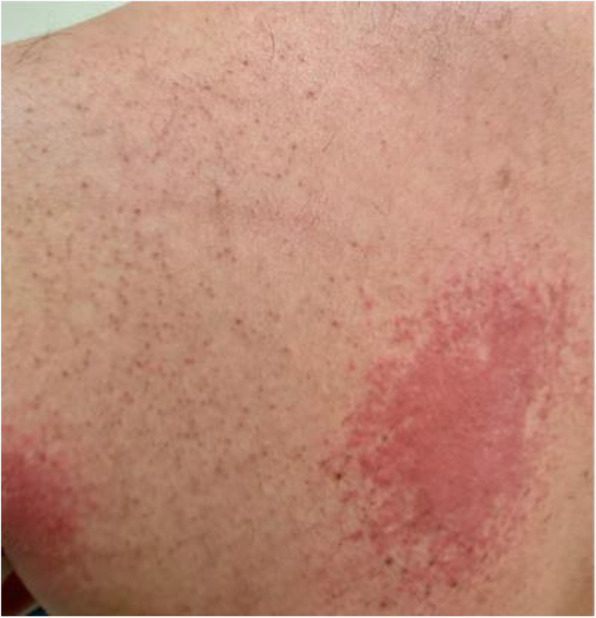
Rash on patient’s back

His integumentary examination revealed erythematous, raised rough warm plaques on the anterior left lateral chest wall that extended to the medial aspect of the left axilla and mid-thoracic area on his back left to the mid-line (Figs. 1 and 2).

Investigations showed positive results for point of care influenza B swab test and unremarkable full blood count, renal profile and C-reactive protein levels. A throat culture was also sent.

He received 1.2 million units of intramuscular (IM) penicillin G benzathine under the clinical impression of scarlet fever, intravenous (IV) diphenhydramine and methylprednisolone as symptomatic treatment and paracetamol for fever. He was then discharged on oral oseltamivir, loratadine, and paracetamol and was advised to continue using the topical steroid cream.

A day later, the patient reported no improvement and decided to seek medical care again. He was seen in the emergency department with worsening pruritus and pain in the lesion and was referred to the dermatology clinic to be seen on the same day. The dermatologist decided to perform a biopsy on the lesion and discharged the patient on clobetasol 0.05% topical cream for 14 days and oxycodone oral tablets with a follow-up in the dermatology clinic in 14 days.

**Fig. 3:**
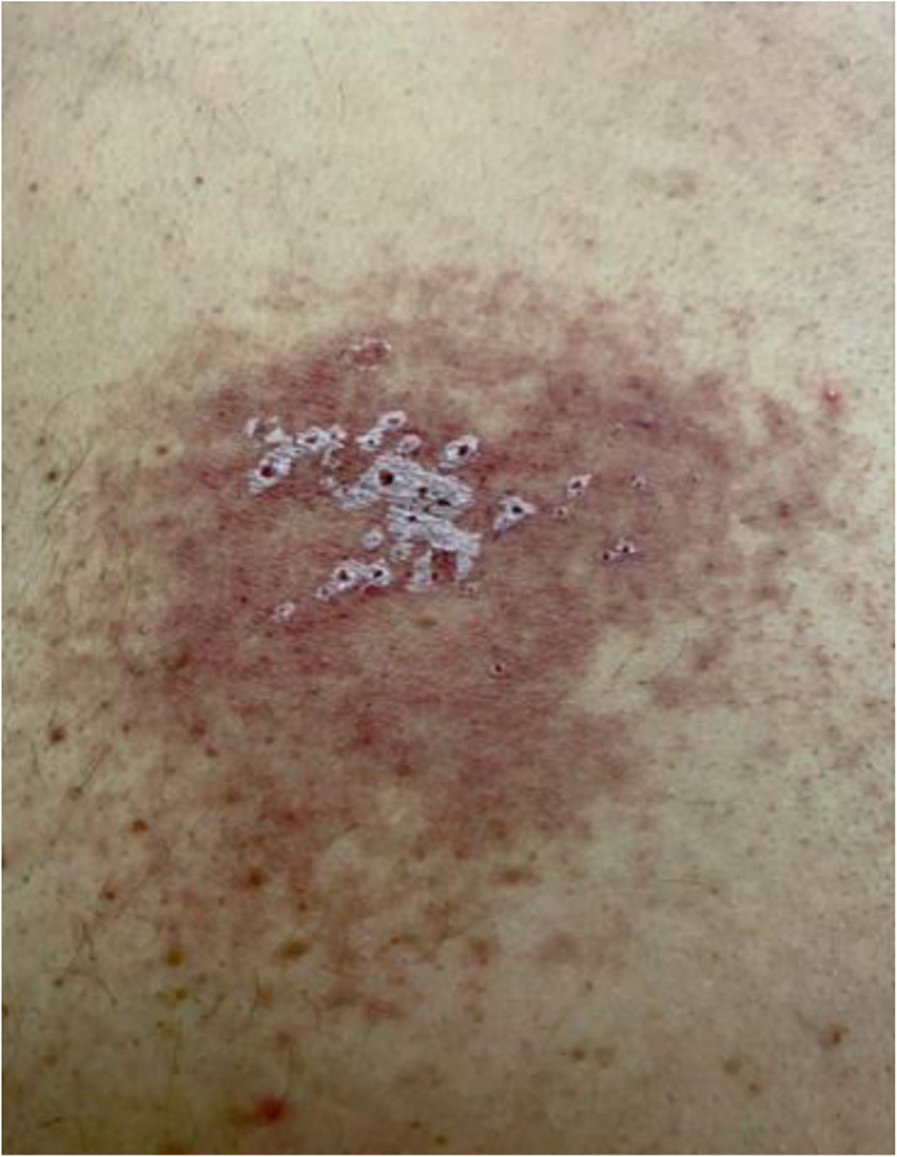
Rash showing white crust that appeared on day 6

Fourteen days later, the patient followed-up with the dermatologist. He described a change in the rash couple of days after he was seen in the clinic. He described raised vesicles that disappeared a week later leaving white lesions that persisted till the day of the follow-up appointment (Fig. 3). His skin biopsy was reported positive for herpes zoster virus. He was then prescribed oral valacyclovir for 7 days with the diagnosis of multidermatomal herpes zoster (T1-T4).

## Discussion and conclusions

Herpes zoster is a unilateral dermatomal disease, which frequently affects a single sensory nerve. A prodromal phase of pain, itch or both typically precedes the skin manifestation. The skin manifestations include an initial erythematous macular phase followed by a vesiculopapular phase that appears within 1–2 days and continues to erupt over another 3–4 days. At this point, the typical clinical picture of HZ is obvious with lesions of all phases being present over a single dermatomal distribution that does not cross the midline.

Apart from the severe pain and itch, HZ seems to have a very benign self-limiting course in the majority of immunocompetent individuals. However, it can present with life-threatening and debilitating long-standing complications in immunocompromised patients and rarely in the immunocompetent population. Those complications include encephalitis, herpes zoster ophthalmicus, retinitis, delayed contralateral hemiparesis, stroke, myelitis, postherpetic neuralgia and postherpetic itch [3].

Antiviral therapy is the cornerstone treatment for HZ. The efficacy of many agents including acyclovir, valacyclovir and famciclovir has been well-established in multiple meta-analyses. Antiviral therapy was demonstrated to be effective in shortening the viral shedding period, reducing the zoster-associated pain severity and duration, halting new lesion formation and hastening the healing of skin lesions [3, 4]. Additionally, some studies showed a reduction in the incidence of postherpatic neuralgia [5]. The effects of antiviral therapy are most favourable if given during the viral replication time which is ≤ 72 h after onset of the skin rash. Nevertheless, it remains a controversy whether antiviral treatment beyond the 72 h of the onset of rash is beneficial [3]. On the other hand, observational studies showed similar benefits when antivirals were used > 72 h after onset of the skin rash [6, 7]. It is also recommended by expert guidelines to initiate antiviral treatment for patients who presents > 72 h of skin rash onset given they present with ongoing new vesicle formation or presenting with HZ complications as those categories are believed to have active viral replication and hence believed to benefit from the antiviral treatment [1]. The patient in our case study presented beyond the 72-h window; however, he had an ongoing vesicular rash. Therefore, he was initiated on the antivirals by the dermatologist.

In around 16% of patients with herpes zoster, the rash disseminates beyond one dermatome, especially in elderly and immunocompromised individuals. Multidermatomal involvement is uncommon in HZ and usually is indicative of immunosuppression [8]. Rarely, this appears in multiple adjacent dermatomes. Only a few cases of multidermatomal Herpes Zoster have been reported in the literature. Furthermore, only a handful number of cases with this manifestation were described in healthy immunocompetent individuals [9, 10]. A recent case report by Beuerlein et al. [11] summarised the published cases of multidermatomal HZ and found only 9 cases in the literature. Most of the 9 cases described were in patients with known immunodeficiencies such as HIV and various malignancies with around 66% involving cervical dermatomes.

Despite the fact that herpes zoster remains a clinical diagnosis, the delay in this patient’s diagnosis is thought to be due to two main factors. The first factor is the atypical presentation with multidermatomal involvement. The second factor is the late onset of the typical vesicular eruption. This has been described in some previous case reports where pain precedes the skin manifestation by more than 7 days or even 3 weeks [5].

We concluded that painful skin manifestation should alert the clinician to broaden the differential diagnosis to include and consider herpes zoster and its prodromal phase [12].

## Abbreviations

ED: Emergency department
HZ: Herpes zoster
IV: Intravenous
IM: Intramuscular
